# Supplementary material for: Transcription-independent regulation of STING activation and innate immune responses by IRF8 in monocytes
Source: Nat Commun. 2022 Aug 16;13:4822. doi: 10.1038/s41467-022-32401-1 (PMC9381507; doi:10.1038/s41467-022-32401-1)
Supplement: Supplementary file 1 — Supplementary Information [file 41467_2022_32401_MOESM1_ESM.pdf]

Supplementary Information

Regulation of STING activation and innate immune responses by IRF8 independent of its transcriptional role in monocytic cells

Wei-Wei Luo[1], Zhen Tong[1], Pan Cao[2], Fu-Bing Wang[3], Ying Liu[2], Zhou-Qin Zheng[1], Su-Yun Wang[1], Shu Li[2,3], Yan-Yi Wang[1]*

*Corresponding Author: Dr. Yan-Yi Wang, Email: wangyy@wh.iov.cn

This PDF file includes:

Supplementary Figures 1 to 6

Supplementary Table 1 to 4

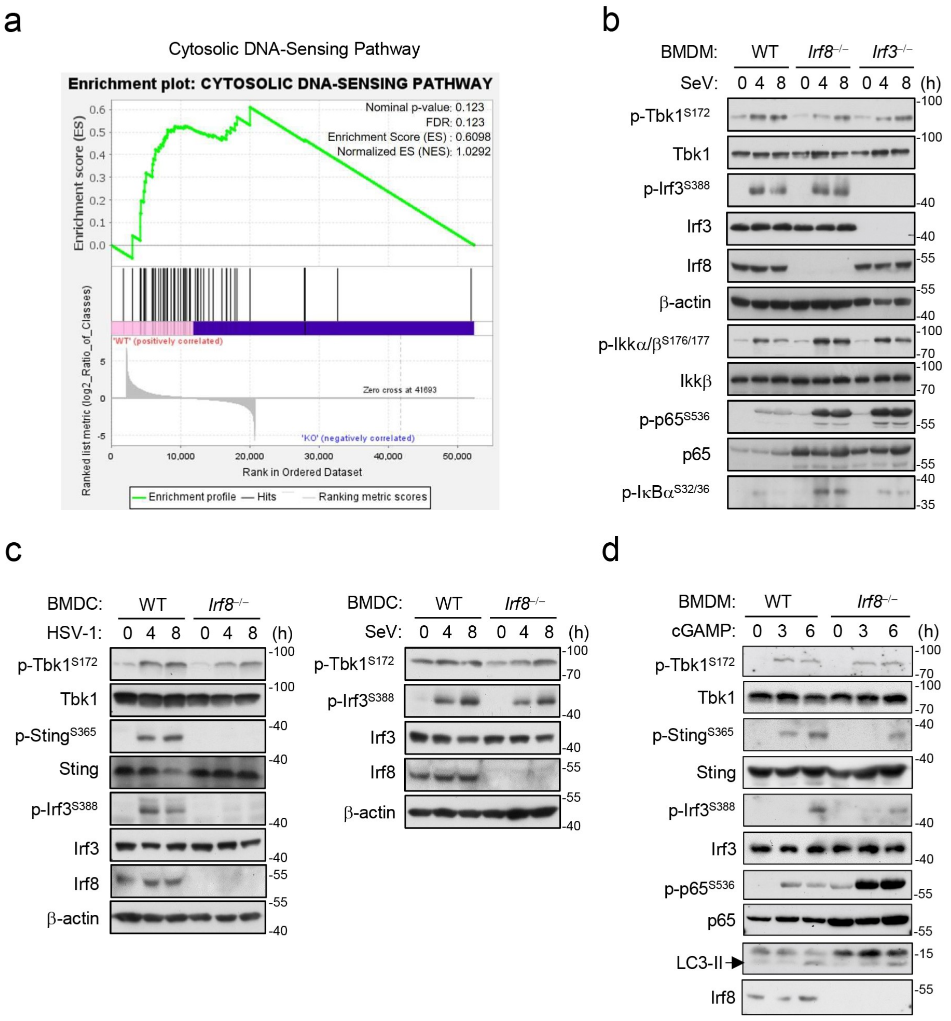

Supplementary Figure 1. Irf8-deficiency does not inhibit SeV-triggered signaling in monocytes.

a, Gene Set Enrichment Analysis (GSEA) enrichment score curves of cytosolic DNA-sensing pathway in WT vs. *Irf8*$^{-/-}$ BMDMs.

b, Immunoblotting analysis of the indicated proteins in WT, *Irf8*$^{-/-}$ or *Irf3*$^{-/-}$ BMDMs un-infected or infected with SeV for the indicated times.

c, Immunoblot analysis of the indicated proteins in WT and *Irf8*$^{-/-}$ BMDCs un-infected or infected with HSV-1(left) or SeV (right) for the indicated times.

d, Immunoblotting analysis of the indicated proteins in WT and *Irf8*$^{-/-}$ BMDMs un-treated or treated with cGAMP for the indicated times.

Source data are provided as a Source data file.

Supplementary Figure 2

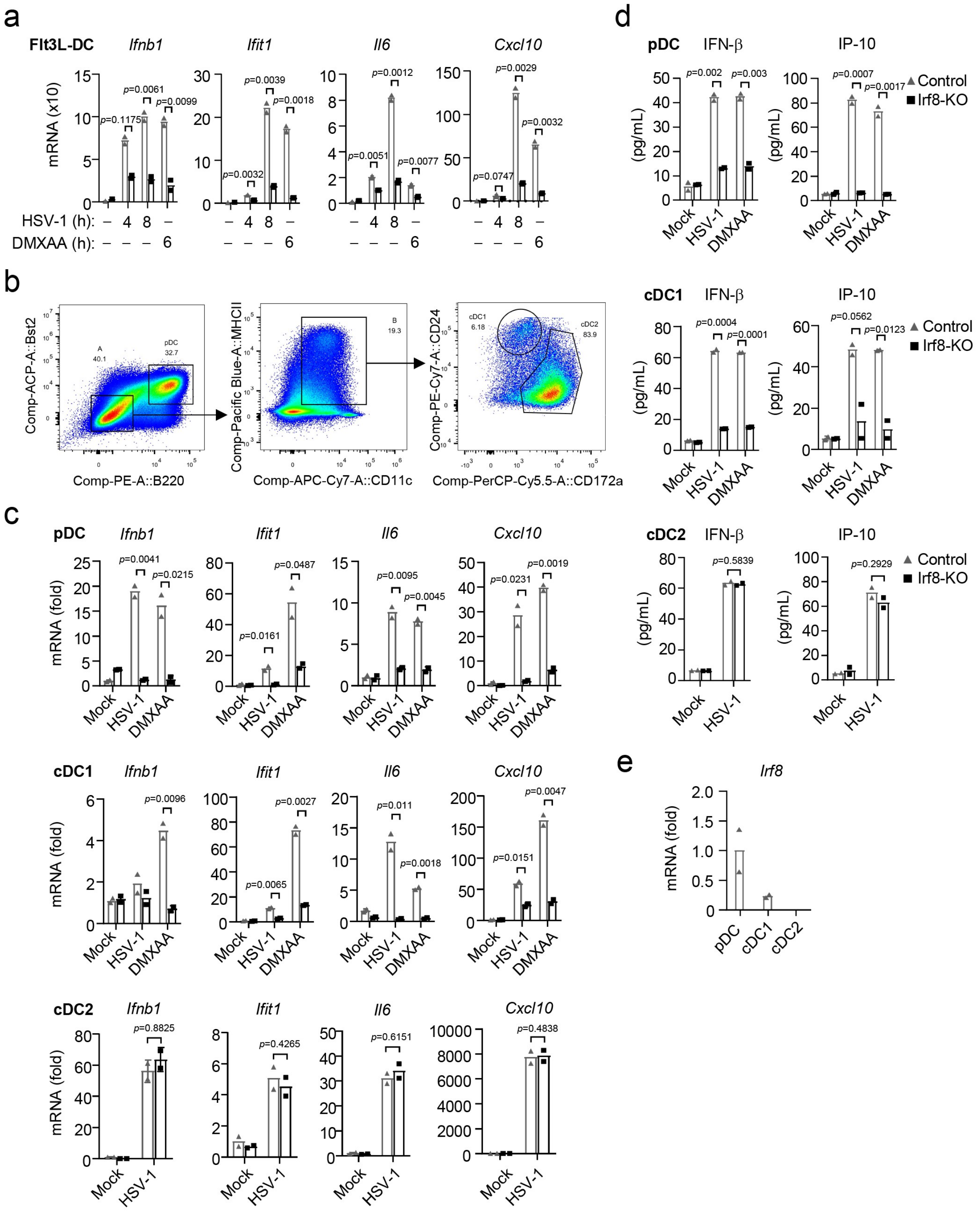

Supplementary Figure 2. Irf8 regulates Sting-mediated innate immune responses independent of its role in monocytic cell differentiation.

a, qPCR analysis of *Ifnb1*, *Il6*, *Ifit1* and *Cxcl10* mRNA levels in Flt3L-BMDCs infected with HSV-1 or treated with DMXAA (50 μg/mL) for the indicated times.

b, Gating strategy to sort pDC ($Bst2^{-}$ $B220^{+}$), cDC1 ($Bst2^{-}$ $B220^{-}$ $CD11c^{+}$ $MHCII^{+}$ $CD24^{+}$ $CD172a^{-}$) and cDC2 ($Bst2^{-}$ $B220^{-}$ $CD11c^{+}$ $MHCII^{+}$ $CD172a^{+}$) cells from C57BL/6 WT mice.

c, qPCR analysis of Ifnb1, Il6, Ifit1 and Cxcl10 mRNA levels in the indicated cells infected with HSV-1 or treated with DMXAA (50 μg/mL) for the indicated times.

d, ELISA analysis of Ifn-β and IP-10 secretion in the indicated cells infected with HSV-1 or treated with DMXAA (50 μg/mL) for 6 hours.

e, qPCR analysis of *Irf8* mRNA levels in the indicated cells.

Data in a, c-e are presented as mean, n = 2 biological replicates. Data were analyzed by unpaired two-tailed Student’ s t-test. Source data are provided as a Source data file.

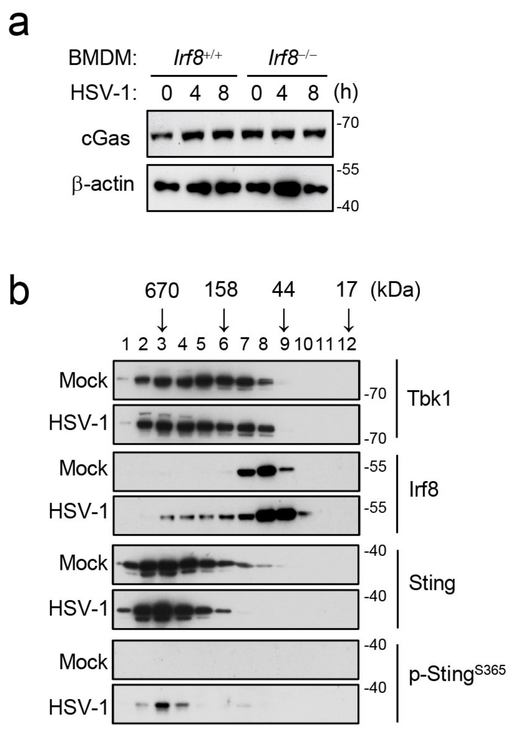

Supplementary Figure 3. **Irf8 acts at the Sting level.**

a, Immunoblot analysis of the expression of cGas in WT and *Irf8*$^{-/-}$ BMDMs un-infected or infected with HSV-1 for the indicated times.

b, HSV-1 infection induces complex formation of Irf8, Sting and Tbk1. BMDMs were left uninfected or infected with HSV-1 for 4 hours. Cell lysates were then fractionated by gel filtration chromatography, and the individual fractions were analyzed by immunoblotting with the indicated antibodies. Fraction sizes were calibrated with the gel filtration standard (Bio-Rad 151-1901).

Source data are provided as a Source data file.

Supplementary Figure 4

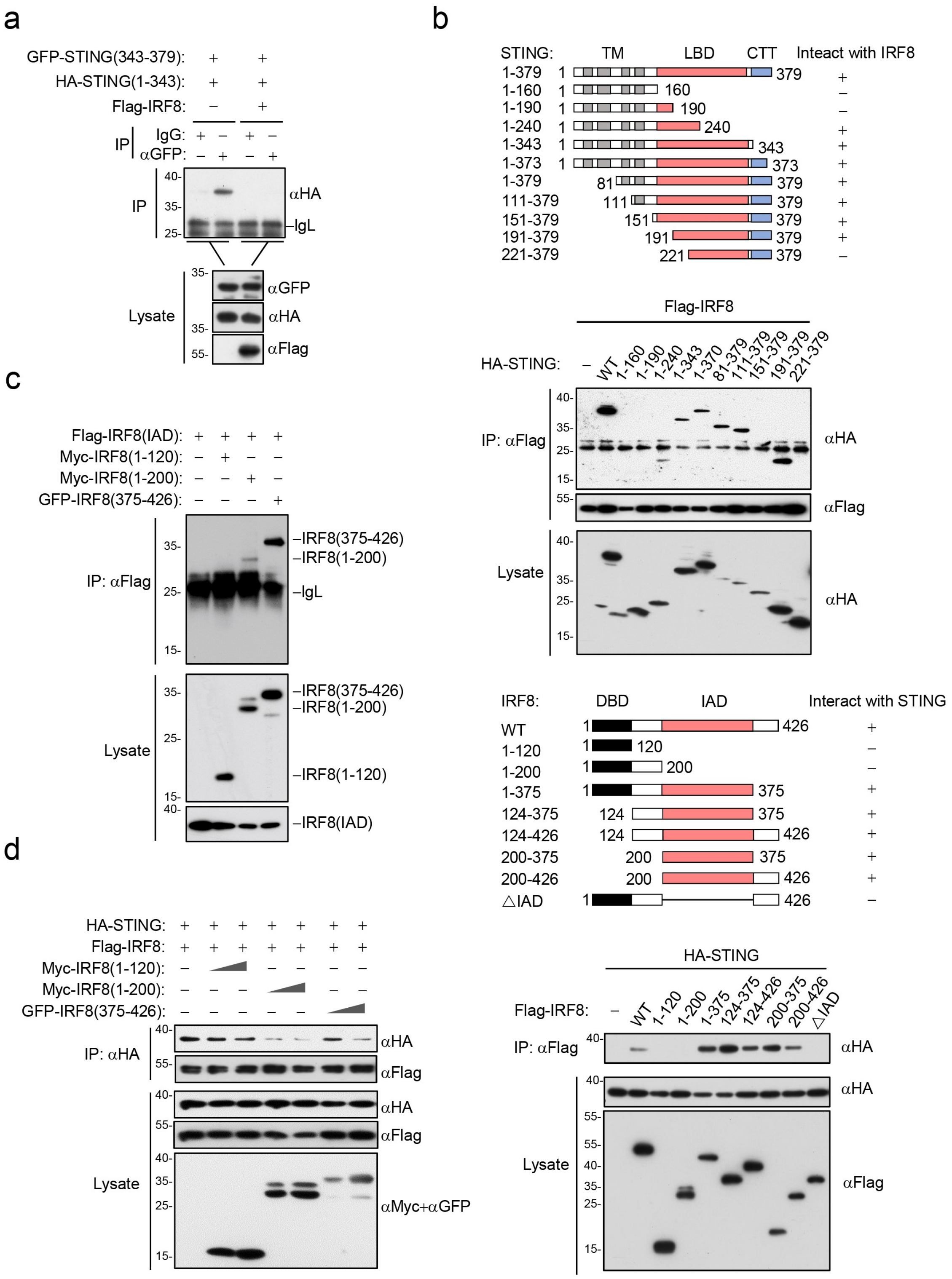

Supplementary Figure 4. IRF8 exists in an auto-inhibitory status.

a, Effects of IRF8 on auto-inhibition of STING. HEK293T cells were transfected with the indicated plasmids for 24 hours before co-immunoprecipitation and immunoblotting analysis with the indicated antibodies.

b, Domain mapping of IRF8 and STING interaction. HEK293 cells were transfected with the indicated truncations before coimmunoprecipitation and immunoblotting analysis with the indicated antibodies. The results were schematically presented (top panel).

c, Interaction of IRF8 IAD with its N- and C-terminal domains. HEK293 cells were transfected with the indicated plasmids for 24 hours before coimmunoprecipitation and immunoblotting analysis with the indicated antibodies.

d, Effects of N- and C-terminus of IRF8 on its association with STING. HEK293T cells were transfected with the indicated plasmids for 24 hours before coimmunoprecipitation and immunoblotting analysis with the indicated antibodies.

Source data are provided as a Source data file.

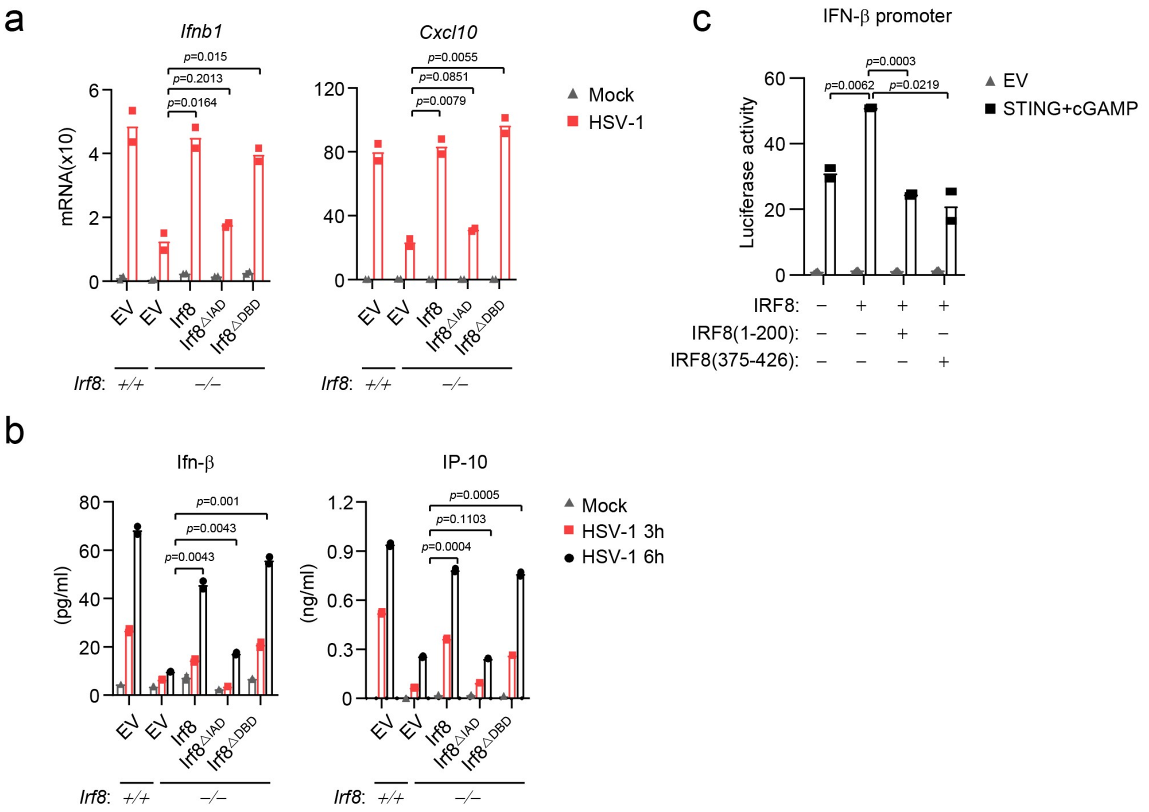

Supplementary Figure 5. The IAD of Irf8 is necessary for Sting-mediated signaling.

a and b, qPCR analysis of Ifn-β and IP10 mRNA levels (**a**) or ELISA analysis of Ifn-β and IP-10 secretions (**b**) in wild-type and *Irf8*$^{-/-}$ BMDMs reconstituted with the indicated plasmids and infected with HSV-1 for 4 hours (**a**) or the indicated times

c, Reporter assays for *IFNB1* promoter activity in HEK293 cells transfected with the indicated plasmids for 24 hours and then treated with 2′3′-cGAMP for 10 hours. EV, empty vector.

Data are presented as mean, (a-b) n = 2 biological replicates, (c) n = 2 technical replicates. Data were analyzed by unpaired two-tailed Student’ s t-test. Source data are provided as a Source data file.

Supplementary Figure 6

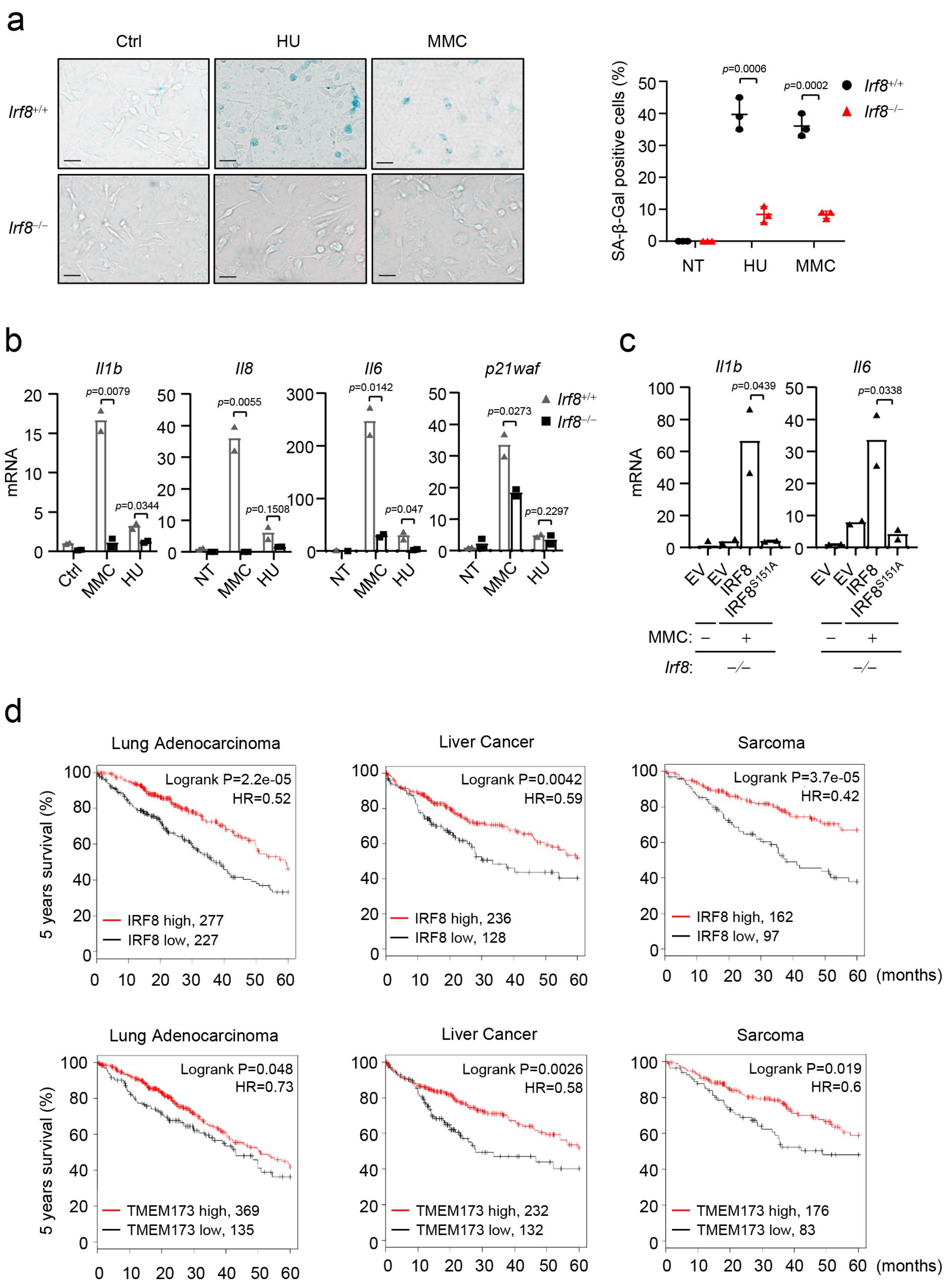

Supplementary Figure 6. IRF8 is involved in DNA damage-induced senescence and tumorigenesis.

a, SA-β-Gal assays for WT and *Irf8*$^{-/-}$ BMDMs treated with hydroxyurea (HU, 10 mM) or mitomycin C (MMC, 1μM) for 5 days. β-galactosidase staining positive cells (blue) in at least three randomly selected fields were counted under a microscope (right). Scale bars, 200 μm. Graph shows mean ± SEM, n=3 independent samples.

b, qPCR analysis of the SASP genes in WT and *Irf8*$^{-/-}$ BMDMs treated with hydroxyurea (HU, 10 mM) or mitomycin C (MMC, 1 μM) for 6 days. NT, untreated.

c, qPCR analysis of *Il1b* and *Il6* mRNA levels in wild-type and *Irf8*$^{-/-}$ BMDMs reconstituted with the indicated plasmids and treated with mitomycin C (MMC, 1μM) for 6 days.

d, IRF8 and STING levels were correlated with beneficial prognosis of cancer patients. Kaplan–Meier survival analysis of IRF8 (top) and STING/TMEM173 (low) in the indicated patients with lung adenocarcinoma, liver cancer or sarcoma. (http://kmplot.com/analysis/).

Data in b-c are presented as mean, n = 2 biological replicates. Data were analyzed by unpaired two-tailed Student' s t-test. Source data are provided as a Source data file.

Supplementary Table 1. A list of DNA oligonucleotides.

VACV70	5′-CCATCAGAAAGAGGTTTAATATTTTTGTGAGACCATCGAAGAGA GAAAGAGATAAAACTTTTTTACGACT-3′
HSV120	5′-AGACGGTATATTTTTGCGTTATCACTGTCCCGGATTGGACACGG TCTTGTGGGATAGGCATGCCCAGAAGGCATATTGGGTTAACCCCTT TTTATTTGTGGCGGGTTTTTTGGAGGACTT-3′
DNA90	5′-TACAGATCTACTAGTGATCTATGACTGATCTGTACATGATCTAC ATACAGATCTACTAGTGATCTATGACTGATCTGTACATGATCTACA -3′
ISD:	5′-TACAGATCTACTAGTGATCTATGACTGATCTGTACATGATCTAC A-3′.

Supplementary Table 2. A list of primary antibodies used in the study.

Antibody	Supplier	Catalog No.	Appl.[a]	Usage
Mouse Flag M2 antibody clone M2	Sigma-Aldrich	#F3165 Lot: SLCC4005	WB/IP	1:2000/1 μg
Mouse HA. 11 Epitope Tag antibody clone 16B12	BioLegend	#901515 Lot: B29401	WB/IF/IP	1:2000/1:200/ 1 μg
Mouse Myc-Tag antibody clone 9B11	CST	#2276S Lot: 24	WB	1:2000
Mouse β-actin antibody clone AC-74	Sigma-Aldrich	#A2228 Lot: 099M47762	WB	1:5000
Mouse β-tubulin antibody	ABclonal	#AC021 Lot: 9100010002	WB	1:5000
Mouse ICSBP/IRF8 antibody(E-9)	Santa Cruz	SC-365042 Lot: D1917	WB/IF	1:1000/1:200
Mouse GFP antibody(B-2)	Santa Cruz	SC-9996 Lot: #G3019	WB	1:2000
Rabbit TBK1 antibody	Abcam	ab109735	WB	1:1000
Rabbit phosphor-TBK1 (S172) antibody	CST	#5483S Lot: 13	WB	1:1000
Rabbit cGAS antibody	CST	#31659S Lot: 3	WB	1:1000
Rabbit STING antibody	CST	13647S Lot: 5	WB	1:1000
Rabbit phosphor-STING (S366) antibody	CST	19781S Lot: 1	WB	1:1000
Rabbit phosphor-STING (S365) antibody	CST	#62912S Lot: 1	WB/IF	1:1000/1:100
Rabbit phosphor-IRF3 (S396) antibody	CST	4947S Lot: 13	WB	1:500
Rabbit IκBα antibody	CST	4812S Lot: 11	WB	1:1000
Mouse phosphor-IκBα (S32/36) antibody	CST	9246L Lot: 19	WB	1:1000
Rabbit p65 antibody	CST	8242S Lot: 9	WB	1:2000
Rabbit phosphor-p65 (S536) antibody	CST	3033S Lot: 17	WB	1:2000
Rabbit IKKβ antibody	CST	8943S Lot: 4	WB	1:1000
Rabbit phosphor-IKKα (S176)/β (S177) antibody	CST	2078S Lot: 9	WB	1:1000
Rabbit LC3B antibody	Abcam	ab192890 Lot: GR-3338049-25	WB	1:1000
Rabbit STAT1 antibody(E-23)	Santa Cruz	SC-346 Lot: #K1115	WB	1:1000
Rabbit IRF3 antibody	Proteintech	11312-1-AP Lot:00082872	WB	1:1000

Supplementary Table 3. A list of gRNA sequences.

Human IRF8-gRNA-1	5′-GTACAGTGCGACAGCTAGAAT-3′
Human IRF8-gRNA-2	5′-GTGACCGGAATGGTGGTCGG-3′

Supplementary Table 4. A list of qPCR sequences**.**

Actin Forward	5′-CATTGCTGACAGGATGCAGAAGG-3′
Actin Reverse	5′-TGCTGGAAGGTGGACAGTGAGG-3′
Ifnb1 Forward	5′-TCCTGCTGTGCTTCTCCACCACA-3′
Ifnb1 Reverse	5′-AAGTCCGCCCTGTAGGTGAGGTT-3′
Cxcl10 Forward	5′-ATCATCCCTGCGAGCCTATCCT-3′
Cxcl10 Reverse	5′-GACCTTTTTTGGCTAAACGCTTTC-3′
Il1b Forward	5′-ACGGACCCCAAAAGATGAAG-3′
Il1b Reverse	5′-TTCTCCACAGCCACAATGAG-3′
Il8 Forward	5′-GTCCTTAACCTAGGCATCTTCG-3′
Il8 Reverse	5′-TCTGTTGCAGTAAATGGTCTCG-3′
Il6 Forward	5′-TCTGCAAGAGACTTCCATCCAGTTGC-3′
Il6 Reverse	5′-AGCCTCCGACTTGTGAAGTGGT-3′
p21waf Forward	5′-CAGATCCACAGCGATATCCAG-3′
p21waf Reverse	5′-AGAGACAACGGCACACTTTG-3′
GAPDH Forward	5′-GACAAGCTTCCCGTTCTCAG-3′
GAPDH Reverse	5′-GAGTCAACGGATTTGGTGGT-3′
IFNB1 Forward	5′-TTGTTGAGAACCTCCTGGCT-3′
IFNB1 Reverse	5′-TGACTATGGTCCAGGCACAG-3′
IFIT1 Forward	5′-TCATCAGGTCAAGGATAGTC-3′
IFIT1 Reverse	5′-CCACACTGTATTTGGTGTCTACG-3′
CXCL10 Forward	5′-GGTGAGAAGAGATGTCTGAATCC-3′
CXCL10 Reverse	5′-GTCCATCCTTGGAAGCACTGCA-3′
Il12b Forward	5′-ATGGCCATGTGGGAGCTGGAG-3′
Il12b Reverse	5′-TTTGGTGCTTCACACTTCAGG-3′
